# Totally endoscopic and video-assisted mitral valve repair: Complementary approaches within a comprehensive surgical program

**DOI:** 10.1016/j.xjon.2026.101620

**Published:** 2026-02-07

**Authors:** Samer Kassem

**Affiliations:** Department of Cardiovascular Surgery, Centro Cardiologico Monzino IRCCS, Milan, Italy

**Keywords:** mitral valve repair, endoscopic, video-assisted

**Figure:**
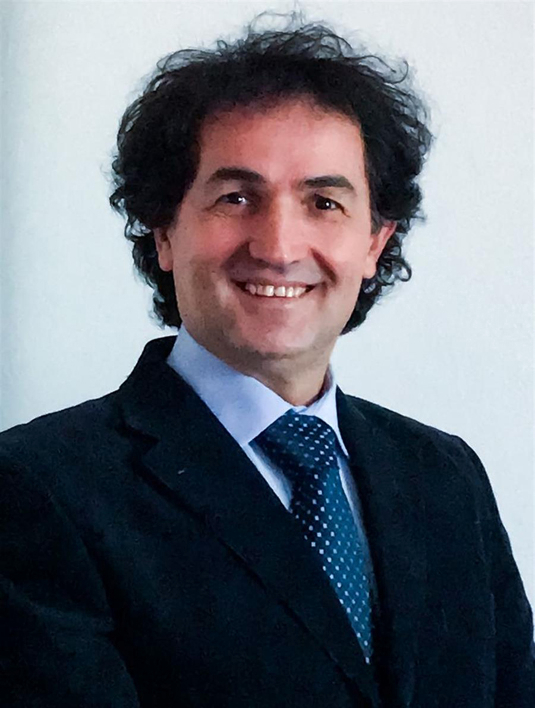
Dr Samer Kassem, MD, PhD

**Central Message**

The use of endoscopic assistance in minimally invasive mitral surgery is not mandatory for good outcomes, but it enhances visualization and facilitates complex reparative maneuvers.

Minithoracotomy in valvular surgery has increased in recent years and is often preferred by patients for aesthetic reasons and faster recovery, factors that influence communication from centers and therapeutic decisions.^1^ However, the concept of minimally invasive cardiac surgery remains primarily related to the size of the skin incision and the avoidance of sternotomy, whereas the use of cardiopulmonary bypass remains indispensable in the treatment of open-heart valvular diseases, unlike in transcatheter approaches.^2^

Comparison between direct vision and optical support suggests that forgoing endoscopic assistance does not compromise the quality of mitral surgery, with comparable clinical and technical outcomes across different approaches.^3^ This perspective reflects an interpretation of currently available published data and aims to address conceptual and organizational aspects of minimally invasive mitral surgery, acknowledging that further evidence will be required to refine these concepts in future studies.

However, published series show differences in repair strategies between totally endoscopic and video-assisted approaches. In totally endoscopic mitral valve repair (122 patients), annuloplasty combined with nonresective techniques predominates.^4^ In contrast, video-assisted minithoracotomy repair (309 patients) demonstrates a more balanced use of resect and respect techniques, with a broader spectrum of reparative maneuvers.^5^

This difference does not reflect lower efficacy of totally endoscopic surgery but rather a greater tendency toward standardized strategies, whereas optical support appears to facilitate the execution of more complex reparative maneuvers that adhere more closely to the classical principles of mitral valve repair.

In reference centers, minimally invasive mitral surgery should therefore be organized as a structured and integrated program, incorporating both totally endoscopic and video-assisted approaches. These techniques should be considered complementary rather than alternative, allowing appropriate management of varying anatomical and reparative complexities.

Mastery of both techniques is essential to enable personalized selection of the surgical approach while preserving the integrity of the reconstructive gesture and the principles of mitral valve repair. Access selection should be guided by valve anatomy, reparative strategy, and patient characteristics, rather than by the degree of minimally invasiveness alone. At the same time, surgeon expertise and familiarity with the chosen platform represent a key determinant of outcomes, possibly more relevant than the approach itself. Different techniques should therefore be viewed as tools to be selected according to individual skills and institutional experience rather than as competing paradigms.

Operating exclusively while looking at a screen should not be presented as a superior skill. In mitral surgery, surgical value lies in anatomical judgment and the quality of the reconstructive maneuver, not in the viewing modality. Technologies, including endoscopic optical systems, should support visualization and decision-making without replacing surgical experience, with the aim of improving patient safety and repair quality.

Conversely, a minimally invasive approach without optical support, based solely on the surgeon's direct vision, limits sharing of the operative field. In this context, only the primary operator has a complete view of intrathoracic events, while the assistant, an essential contributor to procedural safety and effectiveness, is effectively excluded from technical and decision-making processes. The use of a head-mounted camera allows transmission of the intrathoracic view to the team; however, movements of the surgeon's head render the image unstable and intermittent, limiting the ability of the team to continuously follow the surgical gesture and anticipate critical steps or potential procedural complexities. Moreover, the head-mounted camera provides a view concentrated along a “cylinder” extending from the thoracic wall toward the area of interest, reducing perception of the peripheral operative field and making it difficult to identify parietal lacerations caused by retraction.

In conclusion, the balanced integration of new technologies, including optical systems, should be oriented toward patient benefit and institutional care quality rather than individual visibility. Technology should strengthen team cohesion, promote shared understanding of the procedure, and provide access to the most appropriate methods to achieve durable and effective mitral valve repair. Institutional review board approval and patient consent were not required for this article.

## Conflict of Interest Statement

The author reported no conflicts of interest.

The *Journal* policy requires editors and reviewers to disclose conflicts of interest and to decline handling or reviewing manuscripts for which they may have a conflict of interest. The editors and reviewers of this article have no conflicts of interest.
